# Improving Health-Related Quality of Life among People Living with HIV: Results from an Impact Evaluation of a Food Assistance Program in Uganda

**DOI:** 10.1371/journal.pone.0135879

**Published:** 2015-08-27

**Authors:** John A. Maluccio, Tia Palermo, Suneetha Kadiyala, Rahul Rawat

**Affiliations:** 1 Department of Economics, Middlebury College, Middlebury, Vermont, United States of America; 2 Program in Public Health, Stony Brook University (SUNY), Stony Brook, New York, United States of America; 3 Department of Population Health, London School of Hygiene and Tropical Medicine, London, United Kingdom; 4 Poverty, Health, and Nutrition Division, International Food Policy Research Institute, Dakar, Senegal; UNAIDS, TRINIDAD AND TOBAGO

## Abstract

**Introduction:**

Widespread food insecurity in Africa continues to compromise an effective response to the AIDS epidemic. Health-related quality of life (HRQoL) is a comprehensive indicator of physical, mental, and social well-being that is associated with food insecurity and increasingly used to assess the well-being of people living with HIV/AIDS (PLHIV). We examined the impact of a food assistance intervention, previously shown to have reduced household food insecurity and improved nutritional status, on HRQoL of PLHIV.

**Methods:**

We capitalized on an existing intervention targeting antiretroviral therapy (ART)- naïve PLHIV in Uganda, and conducted a prospective impact evaluation including a treatment and a comparison group. Data analyzed included 640 participants from two districts (318 in the intervention district) interviewed in both clinic and household settings at baseline and again approximately one year later. The main outcomes considered were physical and mental health dimensions of HRQoL, and other outcomes included self- and healthcare provider-reported symptoms. We utilized difference-in-difference propensity score matching methodologies to infer causality and examine program impacts.

**Results:**

Over 12 months, food assistance significantly increased physical health scores (PHS) by 2.85 (P < .01) or approximately 0.35 SD, and reduced substantially the number of self- and healthcare provider-reported HIV-related symptoms by 3.83 and 2.68, respectively (P < .01). There was no significant impact, however, on mental health scores (MHS).

**Conclusions:**

This study demonstrates the potential importance for HRQoL of including food assistance programming as part of the standard of care for PLHIV in areas of widespread food insecurity.

## Introduction

HIV/AIDS exacerbates food insecurity through its effects on the productivity of prime working-age adults and, in a mutually reinforcing cycle, food insecurity diminishes the health and welfare of people living with HIV (PLHIV). Prevalent in Uganda and other regions of sub-Saharan Africa, especially among PLHIV [1,2], food insecurity is associated with nutrient inadequacy [3], poor self-reported health [4,5], high-risk behaviors [6], cardiovascular risk factors and diabetes [7], and mortality [8]. Moreover, it has been shown to compromise retention in care and treatment programs, as well as adherence to anti-retroviral therapy (ART) [9,10]. Consequently, improving food security is recognized as fundamental across the four pillars—prevention, care, treatment, and mitigation—of a holistic response to the AIDS epidemic [11–13].

With this recognition has come increased emphasis on food security interventions and growing evidence on their effectiveness. Research has demonstrated that interventions such as food assistance often are able to improve the food security and nutritional status of PLHIV, for example, but generally do not affect immunological outcomes such as CD4 count or disease progression [14–17]. There is minimal evidence, however, on whether food security interventions can improve other important welfare indicators such as health-related quality of life (HRQoL), which captures physical, mental, and social well-being.

This is an important evidence gap as consideration of HRQoL and its role has grown in recent years [9,18–23] and it has been argued that programs incorporating economic strengthening have high potential for improving quality of life [24]. Pozniak [19] argues that recent findings from high-income settings demonstrate PLHIV (including those on ART) typically have lower levels of HRQoL than the general population [18], but that additional research is needed. Furthermore, studies from Africa demonstrate that among PLHIV, worsening HIV disease progression, as measured by lower CD4 count or higher viral loads, is associated with lower HRQoL levels [25–27]. Together, this evidence points to a need for further examination of HRQoL among PLHIV and interventions that can improve it.

A comprehensive measure of well-being that complements biological and anthropometric indicators, HRQoL measures how well a person functions and his or her perceptions (based on experiences, beliefs, and expectations) of physical, mental, and social well-being [28,29]. It is often used to assess the health and well-being of individuals with chronic disease. In an era when improved care and treatment, for example through better management of opportunistic infections, has transformed HIV/AIDS into a chronic condition for millions, HRQoL for PLHIV is increasingly relevant.

Both theory and associational evidence suggest that enhanced food security plausibly might lead to improvements in HRQoL and, more specifically, to improvements in two dimensions that researchers often focus on: physical and mental well-being [9,21,22,30–37]. Conceptually, food and nutrition insecurity might affect HRQoL through various pathways, including nutritional status and chronic stress. Better access to food, and improved nutrition, could lead to improvements in physical functioning and physical health aspects of well-being. Improved food security also could mitigate chronic stress and, consequently, improve mental health aspects of well-being. The potential channel for this latter pathway from food insecurity to chronic stress begins with the observation that in many Sub-Saharan African settings, including Uganda, food insecurity is an important ongoing form of uncertainty experienced in daily living, largely because of the heavy reliance on home food production with all of its accompanying risks and uncertainties. (More than three-quarters of the sample we examine were involved in home agricultural production.) Thus, food insecurity is likely to be strongly associated with daily and chronic stress [38].

Emerging empirical evidence supports these hypothesized pathways. Research using the Medical Outcomes Study (MOS) HIV Health Survey [39] administered to PLHIV sampled from Ugandan clinics found that severe household food insecurity was associated with lower physical HRQoL scores (1.5 to 3.3 points from a mean of 50 and standard deviation 10) and lower mental HRQoL scores (1.7 points) among PLHIV [9,22,40]. Food insecurity also has been found to be associated with poor mental health as measured by depressive symptoms and anxiety [10,37,38,41]. In the single published study to our knowledge investigating whether food insecurity interventions can improve HRQoL among PLHIV, however, Oketch and colleagues found no differences in general quality of life between recipients and non-recipients of non-specific nutrition, care, and support services in South Africa [34].

We capitalized on an existing intervention for ART naïve PLHIV in northern Uganda, coordinated by The AIDS Support Organization (TASO)—an HIV/AIDS care and treatment organization—and the World Food Programme (WFP), to conduct a prospective impact evaluation of a monthly household food basket on the physical and mental dimensions of HRQoL. We hypothesized that food assistance would improve both physical and mental components of HRQoL. Using quasi-experimental methods to identify causal impacts, in this paper we examined whether a food assistance intervention previously shown to have reduced household food insecurity and improved nutritional status [14], also improved HRQoL. The former were the primary programmatic objectives of the food assistance intervention implemented by TASO and WFP examined here; in the current study we turned to an assessment of impacts on secondary objectives of the program.

## Methods

### Study Design

TASO provides comprehensive HIV prevention and AIDS care and support services, including livelihood training and extensive counseling aimed at providing psychosocial support to its clients and their families. In particular, counseling at all stages has been an integral aspect of its work since the organization was founded over two decades ago [42] and the organization promotes a philosophy of “living positively with HIV.” In some areas where it works, TASO partners with WFP to deliver food assistance in the form of monthly food baskets for specifically targeted clients. To evaluate the impacts of this food assistance, we conducted a 12-month prospective impact evaluation nested within the routine programmatic context of both TASO and WFP in two districts in northern Uganda, Gulu and Soroti [14]. The study districts, each with a single TASO clinic, were more than 100 kilometers apart and both were highly food insecure with histories of armed conflict and internal displacement [43]. During the study, WFP operated in Gulu but not in Soroti; thus Soroti served as the non-randomized comparison district.

We recruited HIV-positive non-pregnant adults (aged 18 and over) during their routine visits to their respective TASO clinic who: 1) were eligible for food assistance based on WFP’s poverty assessment criteria but had not received food assistance from any source in the previous 12 months; 2) were ART naïve; and 3) had a CD4 count between 200 and 450 cells/μL. Recruitment procedures were identical across districts. Individuals receiving ART were excluded from the study because of the hypothesized large differential impacts on the primary study objectives among those receiving and those not receiving ART. We focused on those who were not yet eligible for ART (at the time of study initiation), because they were considered one of the most vulnerable populations to food insecurity. Monthly food distribution in Gulu began within 1–4 weeks of recruitment and was conditional on remaining an active TASO client, meeting with a TASO support officer at least once per month.

Multipurpose surveys were administered at baseline and again approximately one year later. Upon recruitment, an individual questionnaire was administered to the study participant in a private room at the TASO clinic by a research interviewer not employed by TASO. Trained and standardized anthropometrists took anthropometric measurements and a TASO laboratory technician drew blood for CD4 count. Within seven days, a research interviewer visited the home of the participant to administer the household questionnaire. The interviewer was trained to maintain strict confidentiality and did not refer to TASO or the HIV status of the study participant during the household interview.

**Fig 1:**
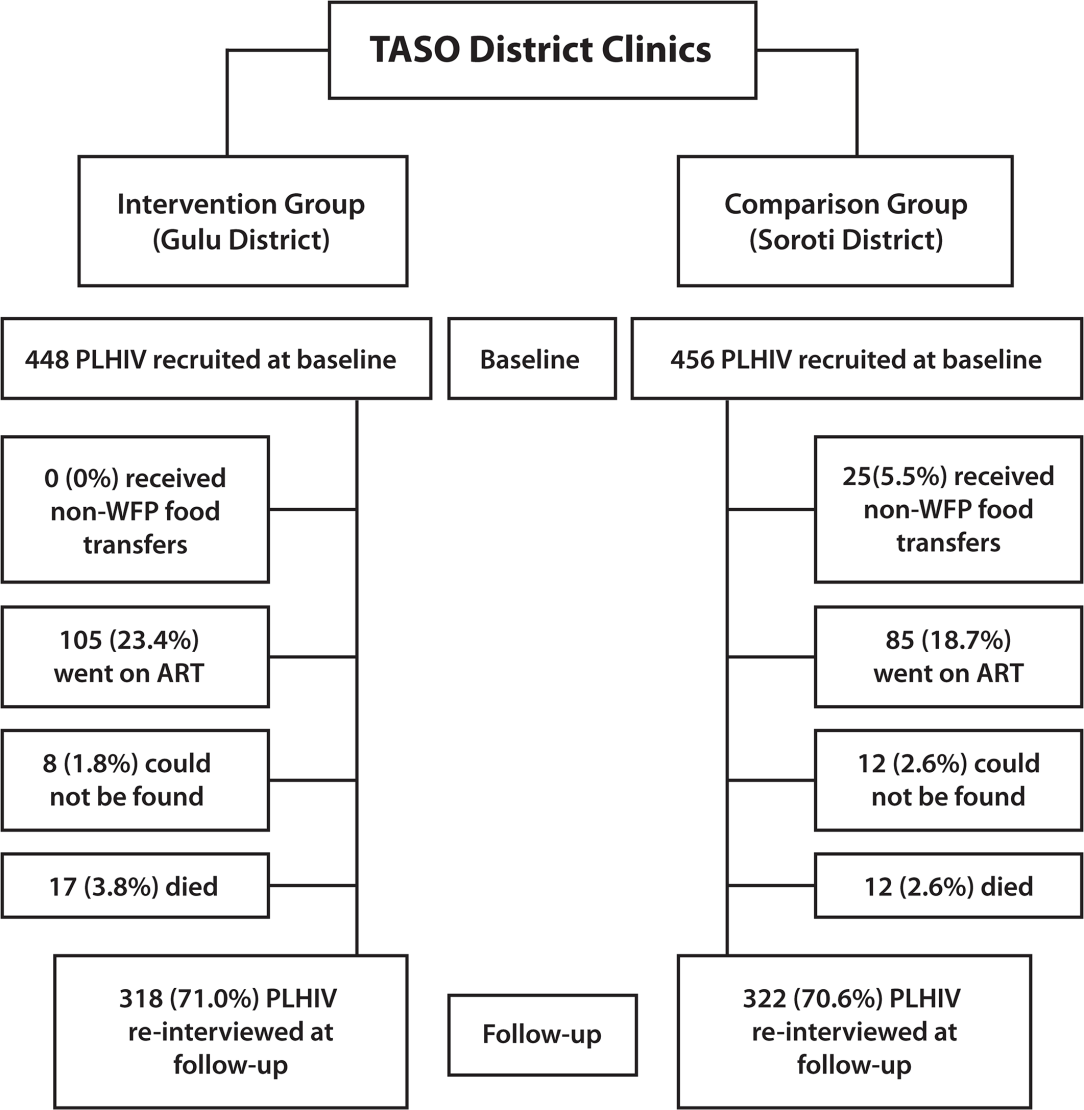
Recruitment and Follow-up of Study Subjects.

The protocol (beginning with the individual survey administered at the clinic and then the household survey at the residence) was the same for the 12-month follow-up interview. Endpoints to the study, for which participants were not re-interviewed, included households in the comparison district that began receiving similar food assistance from another organization after recruitment or individuals in either district initiating ART before follow-up, since the primary outcomes studied in the intervention were nutritional status and CD4 count, both of which could be affected by these developments [14]. The number of individuals becoming eligible for ART after recruitment may have been increased by changes in the World Health Organization (WHO) recommendations for ART eligibility based on CD4 counts made in 2010 [44]. We re-interviewed as many of these individuals as possible *prior* to receipt of outside food assistance or initiation of ART. When such prior interview was not feasible, the individual was considered lost to follow-up (**Fig 1**). Therefore, we did not interview all participants a full 12 months after recruitment—77% of study subjects at follow-up were re-interviewed between 10–13 months after their baseline interview, 15% were interviewed before 10 months (mean 8.2; standard deviation [SD] 1.8) and 8% after 13 months (mean 14.6; SD 2.2).

The ethics review boards of TASO, the Uganda National Council on Science and Technology, and the International Food Policy Research Institute approved the study protocol. Interviewers read consent forms to study participants who provided signed informed consent.

### Measures

We measured HRQoL using a modified version of the MOS-HIV Health Survey [29,45] which has been implemented widely in sub-Saharan Africa [27,34,46,47]. The instrument was first adapted for use in rural Africa in Uganda itself, and has been shown to have good reliability and validity among PLHIV in Uganda and elsewhere [21,27,39,48]. The MOS-HIV survey consisted of 34 questions assessing 10 dimensions of HRQoL, including general health perceptions, physical functioning, role functioning, pain, social functioning, mental health, energy/vitality, health distress, cognitive functioning, and quality of life. Interviewers were provided with translations into the common languages of the study. We scored subscales for each of the ten dimensions and then created the two summary scores as outlined in the MOS-HIV User’s Manual [29,40,49]. Briefly, the subscales were first scored as summated rating scales ranging from 0 to 100, where a higher score indicates better health or functioning. We then standardized all subscale scores to have mean 50 and standard deviation (SD) 10 at baseline in our sample. The summary scores, physical health summary (PHS) and mental health summary (MHS) were developed using factors derived from factor analyses conducted by Revicki et al. [49]. For the PHS score, the subscales for physical functioning, pain, and role functioning contributed most strongly whereas for the MHS score, it was the subscales for mental health, health distress, cognitive functioning, and quality of life. Revicki et al. [49] transformed the summary measures to standardize each to a mean of 50 and SD 10 in their sample, with higher scores indicating better health. For comparability with their and other published work, we applied the same transformation factors used in Revicki et al. [49] to our raw scores (and consequently average summary scores in our sample do not have exact mean 50 and SD 10, even at baseline).

Our main outcomes were the two summary scores, PHS and MHS. We also examined each of the ten subscales, as well as responses to the subset of questions from the MOS-HIV on activities of daily living used to form the physical functioning subscale asked a second time of the respondent during the household interview in each round. We assessed internal consistency of the summary scores and all of the subscales using Cronbach’s alpha.

Other outcomes examined included the total number of HIV-related physical conditions and, separately, symptoms [33,34]. We counted all healthcare provider-reported physical conditions from a pre-defined list of 17 items (including, e.g., opportunistic infections, other illnesses, and medical conditions associated with HIV) indicated in the individual’s current TASO clinical records, updated just prior to each research interview. We also counted all self-reported physical symptoms (in the previous 30 days) associated with HIV from a pre-defined list of 16 items.

Several other individual-, household-, and community-level characteristics at baseline were used in the analyses (**Table 1**). These included a number of individual-level demographic characteristics such as gender, age, education, and marital status. Given the nature of the intervention, which provided food assistance to needy households, we also included indicators of household-level economic well-being such as monthly per capita household food and total consumption (the sum of cash expenditures and respondent imputed value of consumption from own-production [50]), and household size. Distance to the nearest market (in kilometers) was included as it affects food prices and access to food. In addition, we included household-level food insecurity and individual-level dietary quality measures found to be associated with HRQoL in this context [40]. We measured household-level food insecurity with the Household Food Insecurity Access Scale (HFIAS) [51], previously validated in sub-Saharan Africa [52–54]. This scale ranges from 0 to 27 and was constructed from responses to nine questions regarding the past 30 days reflecting three areas: 1) anxiety about food supply; 2) insufficient quality of food supply; and 3) insufficient quantity of food supply. We measured individual-level dietary quality with the validated Individual Dietary Diversity Score (IDDS), based on previous day consumption from nine different food groups [55]. Consideration of an individual-level diet measure was novel, as most existing studies include only household-level measures [9,22], even though allocation within the household can lead some individuals to have better access to food than others. Earlier work demonstrated that the intervention being analyzed here improved household food security but had little impact on individual-level dietary quality using these same measures [14].

**Table 1:** Baseline survey variables used in constructing propensity score for matching.

*Individual background (Source*: *individual survey at clinic)*	
	Male (= 1)^1^
	Had marital or other partner for at least two years (= 1)
	Widow/er (= 1)
	Age (logarithm of number of years)
	Highest grade attained (number of grades)
*Individual measured health status (Source*: *individual survey at clinic)*	
	BMI (kg/m^2^)
	Mid-upper arm circumference or MUAC (mm)
	CD4 count (cells/μL)
*Individual self-reported health status and diet (Source*: *individual survey at clinic)*	
	Little or no pain in previous month (= 1)
	Too ill to work in previous month (= 1)
	Physical health summary score from MOS-HIV (PHS score)^1^
	Mental health summary score from MOS-HIV (MHS score) ^1^
	General health subscale score from MOS-HIV (subscale score)
*Household background (Source*: *household survey at residence)*	
	Household Food Insecurity Access Scale (HFIAS score)^1^
	Household size (number of members)
	Dependency ratio (members aged 0–14 plus members aged 65 and over divided by members aged 15–64)
	Per capita monthly consumption (logarithm of value in Ugandan Shillings [USh])^1^
	Per capita monthly food consumption (logarithm of value in USh)
	Food share of household monthly consumption (fraction)
	Value of assets (logarithm of value in USh)
*Distance from household to (Source*: *individual survey at clinic)*:	
	TASO clinic (km)
	Nearest market (km)
*Survey interview characteristics*	
	Time between baseline and follow-up survey interview (months)^1^
	Calendar quarter of follow-up survey interview (quarters 1 to 4)

Notes: Units and transformation for balancing shown in parentheses.

^1^ Used directly in matching procedure as described in methods section.

In addition to analysis of the entire sample, for the main outcomes we also examined specific subgroups motivated by findings from the literature and the nature of the intervention. In particular, we examined men and women separately, as they have been observed in this and similar contexts to have different levels of HRQoL [26,27,40,47]. We next considered two subgroups for whom we hypothesized larger effects if improvements in food security lead to improvements in HRQoL, those individuals in poorer or more food insecure households at baseline. These were households with per capita food consumption below the sample median and, separately, households evaluated as severely food insecure [51]. And last, since CD4 has been shown to be negatively associated with HRQoL [56–59], we separately considered those with higher CD4 (CD4>350) at baseline.

### Statistical Analysis

To estimate the impact of food assistance on dimensions of HRQoL and the other outcomes, we combined difference-in-difference techniques with the bias-adjusted nearest-neighbor matching estimator proposed by Abadie et al. [60–62], with matching based on the estimated propensity scores. The nearest-neighbor matching methodology performs well when there is dense common support for the two distributions, i.e., when there are many nearby neighbors (or possible matches) for treatment observations, as found in the current study. Thus, we compared the change over time in outcomes for all individuals in the intervention group with the change over time in outcomes for weighted matched individuals from the comparison group, an approach increasingly used in evaluations without randomization to construct a comparable statistical counterfactual group [63].

Although individual-level eligibility criteria were identical across districts (limiting potential selection problems related to differential recruitment across study arms), with only two sites (and without randomization), initial differences between treatment and comparison were possible. Therefore, we identified a set of observable individual-, household-, and community-level characteristics likely to be associated with the outcomes (**Table 1**). These included the actual baseline values of the main outcomes to help ensure that there were no initial differences between matched groups, which could lead to bias if the magnitude of change in the outcome depended on the initial baseline level [60,63]. Using these variables, we constructed a propensity score for each individual, estimating the predicted probability of being in the intervention group (i.e., living in the intervention district), as a function of all of the baseline characteristics listed in **Table 1**with logit regression models. We transformed some variables used in the logit as indicated in the table (e.g., using logarithmic transformations) until there were no statistically significant differences in the mean of each variable across intervention and comparison groups for each 20-percent quantile of the propensity score. (Statistical significance of the difference between intervention and comparison groups for each variable, in each quantile, was assessed via a simple regression of the variable on an indicator for the intervention group for all observations in that quantile, using heteroskedasticity-robust standard errors.) Referred to as balancing, this procedure helps ensure that propensity-score based matches have similar values of the underlying balance variables [64,65].

We then used the predicted propensity score from this final model specification, as well as several other important variables (initial baseline PHS and MHS scores, the logarithm of per capita total consumption, HFIAS, IDDS, and the time in months between baseline and follow-up interviews), to match each individual in the intervention group to the most similar individual, or “nearest neighbor,” of the same gender in the comparison group [60]. The estimated average treatment effect on the treated (ATT), then, is the difference-in-difference in mean outcomes over 12 months for the intervention group compared to the matched comparison group, with more weight given to closer matches as per the bias-adjusted estimator [60]. We implemented a heteroskedasticity-robust variance estimator developed for this matching technique [60].

As with any matching estimator, the validity of this approach relies on the unverifiable assumption that conditional on a set of observable characteristics, outcomes in the untreated group are independent of (and therefore uncorrelated with) treatment status. The use of a number of baseline variables to construct the propensity score, as well as several key variables in addition to the propensity score for matching [60] served to strengthen the validity of the comparison. The specific maintained assumption required for validity differs with alternative sets of matching variables or different matching procedures. Therefore, one can indirectly assess the validity of the approach by examining various alternatives; large differences in results across slight modifications in the matching variables, for example, would suggest that the assumption required for validity may be invalid. We carried out sensitivity analyses on the main outcomes to explore this possibility, including: 1) an alternative set of matching variables using the same procedure but using all of the balancing variables directly in the matching procedure, while continuing to match exactly on gender;; 2) a subsample of the original analysis limited to the set of observations with estimated propensity scores between 0.1 and 0.9 to enhance further the density in overlap; and 3) an alternative estimation procedure using Gaussian kernel matching on the estimated propensity score with bootstrapped standard errors based on 1000 repetitions [65].

All analyses were carried out using Stata version 13.1 (College Station, TX). We set statistical significance at a two-tailed P<0.05.

## Results

Between August 2008 and October 2009, we recruited 904 subjects at the intervention (Gulu) and comparison (Soroti) district TASO clinics (but who resided in more than 100 different villages and urban neighborhoods), and between August 2009 and October 2010 followed up with 640 (**Fig 1; S1 Data**). We did not re-interview individuals who: 1) lived in the comparison district and had been provided food assistance from another program after recruitment; 2) had begun ART; 3) could not be located; or 4) had died. Loss to follow-up was the same across districts (29%) and driven largely by ART initiation, a pre-determined endpoint in the study (see Methods: Study design).

Selected baseline characteristics are presented for the intervention group (A), the full comparison group (B), and the matched comparison group (C) (**Table 2**). The intervention sample comprised 242 (76.1%) women out of 318 persons interviewed at follow-up. Nearly 44% of all individuals were married or in a union and another 38% were widowed. Average age was 38.1 years old and average schooling 4.6 completed grades; only 21% had completed primary school (seven years). Average CD4 count was 355.7 cells/μL. Approximately three-quarters of households in the intervention group were categorized as severely food insecure at baseline, and nearly all the rest as moderately insecure, using categories based on HFIAS [51]. Average household size was 6.4 members and based on per capita consumption fully one-quarter of households were below the national poverty line.

**Table 2:** Comparison of Selected Baseline Characteristics.

	Intervention	Comparison All	Comparison Matched^c^	P-Values	
Baseline Characteristics	*(A) (N = 318)*	*(B) (N = 322)*	*(C) (N = 318)*	*(A vs*. *B)*	*(A vs*. *C)*
*Individual*					
Female, n (%)	242 (76.1%)	216 (67.1%)	242 (76.1%)	0.011	1.000
Had marital or other partner, n (%)	139 (43.7%)	175 (54.4%)	149 (46.7%)	0.007	0.426
Widow/er, n (%)	122 (38.4%)	94 (29.2%)	135 (42.5%)	0.014	0.294
Age (years), mean (SD)	38.1 (9.3)	40.7 (10.0)	38.6 (8.5)	0.001	0.465
Highest grade attained, mean (SD)	4.6 (4.2)	4.4 (4.5)	4.5 (4.1)	0.656	0.818
BMI (kg/m^2^), mean (SD)	20.9 (2.5)	20.3 (2.7)	20.5 (2.9)	0.008	0.118
MUAC (mm), mean (SD)	270.5 (30.8)	265.9 (26.7)	267.4 (29.1)	0.044	0.180
CD4 count (cells/μL), mean (SD)	355.7 (57.3)	351.8 (57.9)	353.0 (57.6)	0.405	0.460
Months between surveys, mean (SD)	11.8 (1.4)	10.7 (2.3)	11.6 (1.1)	<0.001	0.037*
IDDS, mean (SD)	3.7 (1.2)	4.5 (1.2)	3.6 (1.2)	<0.001	0.288
*Household*, mean (SD)					
HFIAS (0–27)	16.3 (4.4)	14.2 (5.1)	16.8 (4.8)	<0.001	0.120
Household size	6.4 (2.6)	6.4 (3.0)	6.7 (2.7)	0.783	0.126
Per capita food consumption (USh)	31,619 (20,747)	24,883 (23,090)	24,662 (14,997)	<0.001	0.886
Per capita consumption (USh)	40,743 (32,966)	55,752 (33,872)	40,481 (21,598)	<0.001	0.906
Distance (km) to:					
TASO clinic	7.8 (9.5)	10.1 (8.3)	9.0 (8.4)	0.001	0.090
Nearest market	1.1 (1.5)	2.2 (1.9)	1.4 (1.4)	<0.001	0.052
*Outcome variables*, mean (SD)					
Physical health score (PHS)	47.0 (7.9)	46.7 (7.3)	46.4 (7.4)	0.648	0.340
Mental health score (MHS)	46.3 (7.1)	47.3 (7.2)	46.6 (6.7)	0.071	0.590
Number of self-reported physical symptoms (0–16)^a^	7.3 (2.8)	7.3 (3.0)	7.4 (2.9)	0.833	0.707
Number of healthcare provider-reported physical conditions (0–17)^b^	1.9 (1.8)	1.9 (1.6)	2.0 (1.6)	0.976	0.233
*Propensity Score*, mean (SD)	0.71 (0.2)	0.33 (0.3)	0.71 (0.3)	<0.001	0.930

a. Self-reported physical symptoms: Includes skin rash, body pains, dizzy/headaches, weakness/fatigue, insomnia, numbness (lack of sensation), reduced or loss of vision, fever, stomach upset, vomit, diarrhea, stomach ache, losing hair, loss of appetite, losing weight, and sunken cheeks.

b. Healthcare provider-reported physical conditions: Includes tuberculosis, malaria, diarrhea, respiratory infections/difficulty breathing, syphilis, oral thrush/oral lesions, oral candidiasis, high fever, skin rash, cough, depression, fatigue, herpes zoster, genital herpes, vaginal candidiasis, weight loss, and vision problems.

c. Includes one matched observation from the comparison group for each observation in the intervention group, based on matching procedure described in statistical analysis section. Comparison group individuals matched to more than one individual in the intervention group are duplicated.

Average baseline characteristics for the overall intervention and matched comparison groups were similar for nearly all of the indicators, consistent with the balancing exercise and supporting the credibility of the matching exercise. Out of 24 variables used in the construction of the propensity score, only one was statistically different when comparing the overall intervention group versus the matched comparison—the time in months between surveys with a difference of 0.2 months or approximately 6 days (P = 0.037). Average PHS and MHS at baseline were 47.0 and 46.3 in the intervention group and 46.4 and 46.6, in the matched comparison group; the differences across groups were less than 0.1 SD and insignificant.

Cronbach’s alpha for the ten subscales used to compute the PHS and MHS for all observations was 0.86 at baseline and 0.89 at follow-up, indicating a high degree of correlation among the underlying dimensions for these measures. Cronbach’s alpha for the components of each of the ten subscales (but one) ranged between 0.80 and 0.88 at baseline (0.80 and 0.93 at follow-up), which was higher than reported for a similar population elsewhere in Uganda [66]. The exception, role functioning comprised of only two questions, had weak internal consistency with Cronbach’s alphas below 0.30. The Cronbach’s alpha for the six physical functioning subscale questions asked a second time during the household interview was 0.72 at baseline and 0.75 at follow-up.

The ATT bias-adjusted difference-in-difference matching estimates are presented in **Table 3**; we present impact estimates based on matching with the complete sample of 318 intervention observations and the subgroups detailed in the methods section. For the sample as a whole, food assistance increased PHS by 2.85 (P<0.01) (relative to the weighted comparison group), approximately 0.35 SD, but did not have a statistically significant effect on MHS (Panel A). These were the result of average PHS increasing over time in both the intervention and weighted comparison groups (though more in the former), while average MHS was virtually unchanged in both districts. Each of the estimated effects on PHS (MHS) for the various subgroups was significant (insignificant). There were slightly larger effects on PHS observed for men and for individuals in households with poor resources as measured by HFIAS and per capita food consumption, though none of these were statistically different from the main effect of 2.85 estimated for the overall sample.

**Table 3:** Difference-in-Difference Average Treatment Effect on the Treated (ATT): Nearest Neighbor Matching Results for Health-Related Quality of Life measures.

	Intervention N	Effect (standard error)		95% confidence interval	
**Panel A: HRQoL Summary scores:**					
Physical health score (PHS)					
All individuals	318	2.85*	(0.81)	[1.3,	4.4]
Women only	242	2.38†	(0.98)	[0.5,	4.3]
Men only	76	4.63*	(1.35)	[2.0,	7.3]
HFIAS severe	226	3.71*	(0.98)	[1.8,	5.6]
Per capita food consumption < median	159	5.54*	(1.20)	[3.2,	7.9]
CD4 > 350	167	2.84*	(0.89)	[1.1,	4.6]
Mental health score (MHS)					
All individuals	318	-0.25	(0.97)	[-2.2,	1.7]
Women only	242	-0.65	(1.19)	[-3.0,	1.7]
Men only	76	0.55	(1.51)	[-2.4,	3.5]
HFIAS severe	226	-0.44	(1.01)	[-2.4,	1.5]
Per capita food consumption < median	159	1.84	(1.37)	[-0.8,	4.5]
CD4 > 350	167	1.55	(1.05)	[-0.5,	3.6]
**Panel B: HRQoL subscale scores:**					
General health perceptions	318	6.75*	(1.79)	[3.2,	10.3]
Social functioning	318	2.58†	(1.12)	[0.4,	4.8]
Mental health	318	-1.64	(2.14)	[-5.8,	2.6]
Energy/vitality	318	-6.62*	(1.47)	[-9.5	-3.7]
Health distress	318	5.01*	(1.53)	[2.0,	8.0]
Cognitive functioning	318	10.25*	(1.32)	[7.7,	12.8]
Quality of life	318	1.80	(1.17)	[-0.5,	4.1]
Role functioning	318	11.60*	(1.39)	[8.9,	14.3]
Pain	318	5.34*	(1.37)	[2.6,	8.0]
Physical functioning	318	3.34*	(1.05)	[1.3,	5.4]
Physical functioning (from HH survey)	318	4.48*	(1.42)	[1.7,	7.3]
**Panel C: Other reported physical outcomes:**					
Self-reported symptoms (number)	318	-3.83*	(0.47)	[-4.7,	-2.9]
Healthcare provider-reported conditions (number)	279	-2.68*	(0.51)	[-3.7,	-1.7]

Notes: Standard error in parentheses, 95% confidence interval in square brackets.

*P < .01

†P < .05. All models match exactly on gender and match on the predicted propensity score and months between surveys. Models in Panels A & C also include baseline HFIAS, IDDS, PHS and MHS scores, and the logarithm of per capita consumption; models in Panel B also include baseline scores for all 10 subscales.

Food assistance significantly increased 7 of 10 subscales, and it significantly reduced one subscale, for energy/vitality (Panel B). There were significant positive effects on all three HRQoL subscales found to be strongly associated with PHS (physical functioning, role functioning, and pain). It had significant positive effects on only two (health distress and cognitive functioning) of the four subscales most strongly associated with MHS, however, with no significant effects on the mental health and quality of life subscales [49].

Results for the physical functioning subscale asked a second time each round, 1–4 weeks later during the household survey at the individual’s home, show similar improvements to the subscale based on the survey completed at the clinic, within one standard error of each other (3.34 [standard error (SE): 1.05] for the individual survey and 4.48 [SE: 1.42] for the household survey).

Last, food assistance decreased the number of reported physical symptoms and conditions. Overall, there were 3.83 (P<0.01) fewer self-reported symptoms as similarly reported in [14], and 2.68 (P<0.01) fewer healthcare provider-reported conditions. These differences reflected reductions of 1.5 SD for each outcome.

## Discussion

Our study is the first to examine prospectively the impacts of a food security intervention on HRQoL, a comprehensive outcome measure for well-being, among PLHIV. Using quasi-experimental matching methods to better infer causality, we tested whether the intervention improved two key aspects of HRQoL, the physical health summary and mental health summary scores. We demonstrated that a food assistance intervention provided to households of ART naïve PLHIV in highly food-insecure northern Uganda, and previously shown to have improved household food security and BMI [14], significantly improved physical, but not mental, HRQoL compared to a matched comparison group receiving otherwise similar HIV care, treatment, and counseling.

At baseline, average PHS in our sample of vulnerable PLHIV was approximately the same as that of a similar Ugandan sample [9], but average PHS and MHS were substantially higher than seen in a Ugandan sample with more advanced disease progression [67], consistent with evidence that HRQoL is negatively associated with disease progression [40,56–59].

The results were as hypothesized with respect to PHS, showing statistically and clinically (greater than 0.35 SD [67]), significant effects. They are in contrast, however, to an observational cross-sectional study examining HRQoL of PLHIV by receipt of food assistance in which there were no positive associations with respect to general health, self-care, and physical domains of HRQoL [34]. The difference in results may be due to our having evaluated a specific intervention as well as to our use of a more suitable comparison group, a prospective design, and a robust non-experimental matching methodology.

The results were not as hypothesized with respect to MHS. In previous work, we demonstrated that HFIAS was associated with both PHS and MHS in the cross-section of the baseline analyzed in this study and hypothesized that food assistance might reduce the mental health component of HRQoL, in particular by reducing chronic stress and anxiety associated with food insecurity [10,38,40,41]. This component of food insecurity, however, does not seem to have been affected by the intervention. At baseline in the current study, 98% of households in the intervention group indicated they had worried in the last 30 days that they would not have enough food, but even with food assistance concern about having enough food was largely unchanged at follow-up (95%). While the food assistance led to a reduction in overall food insecurity as measured by HFIAS for these households and improved the nutritional status of the recipients over the period [14], it did not lead to a significant reduction in anxiety about food supply in this chronically food insecure area, nor in any corresponding improvement in the mental health component of HRQoL.

A second potential explanation for the lack of effect on MHS lies in the comprehensive nature of TASO’s psychosocial support services [42], which were provided in equal measure to both intervention and comparison individuals. This extensive counseling may have diminished the potential effect of food assistance on MHS, which remained constant in both districts over the period, despite worsening disease progression as indicated by falling CD4 counts. Conceptually, however, the equal intensity of psychosocial support in the two districts seems less likely to have diminished the potential effects on (reported) physical health to the same degree.

There are some limitations to our study. First, because the comparisons were not randomized and were drawn from a different district, it is possible that unobserved geographical, sociocultural, or other factors explain part of the observed differences over time between groups. Gulu district, for example, suffered more intensively from conflict during the civil war with higher likelihood of internal displacement [43], though even before matching, the differences across districts in initial PHS and MHS, for example, were small (**Table 2**). We attempted to mitigate potential program selection bias by recruiting subjects into intervention and comparison groups using identical criteria, differencing the outcomes over time (thereby controlling for all district-level, as well as individual- and household-level, time invariant factors that enter the model additively), and including a number of matching variables, many of which capture potentially important differences between the two geographic areas. Further reducing concern about bias introduced by geographic-specific confounders was the fact that sample individuals were not concentrated in small geographic areas within the two districts, with about half of them residing more than 10 km away from the TASO clinic and in more than 100 different villages or urban neighborhoods.

Second, a relatively large proportion of individuals (29%) were lost to follow-up, including 21% because they received ART during the study period, an exclusion criterion in the study. We examined baseline characteristics of individuals lost to follow-up, and found no significant differences across intervention and comparison groups so that attrition across the two groups was not evidently different on observable characteristics. Unsurprisingly, ART-related loss to follow-up was associated with lower initial CD4 counts—individuals with baseline CD4>350 were only half as likely to be lost to follow-up. In addition to inclusion of baseline CD4 in the propensity score prediction in the analyses, we also examined estimated effects for those with baseline CD4>350 (N = 167) and found a nearly identical point estimate for PHS (2.84, P<0.01) and insignificant results for MHS. We interpret this as evidence that selective attrition is not driving our results, since loss to follow-up in this subsample was under 15%.

Third, we intentionally restricted eligibility for this study to vulnerable PLHIV who were TASO clients, qualified for WFP food rations, had CD4 count above 200 but below 450 cells/μL at baseline, and were ART naive. Consequently, our findings may not be generalizable to a broader HIV+ population. However, ART naïve or ineligible populations remain an important subgroup of PLHIVs that warrant study of effective interventions to address some of the health and social consequences of HIV. Large numbers of ART eligible PLHIV do not access ART for a variety of reasons, and only an estimated 36% of the 32.6 million PLHIV in low- and middle- income countries are on ART [68].

Despite these limitations, other aspects of the results increase confidence in the findings. First, there is internal consistency between the impacts on PHS and the component subscales (including the physical functioning subscale asked a second time during the household survey) and self- and healthcare provider-reports of conditions and physical symptoms, as well as with previous findings on nutritional status [14,69]. Second, although not statistically different from analysis on all individuals, subgroup analyses demonstrate larger point estimates of the effects of food assistance on PHS for poorer households with the greatest food insecurity at baseline, increasing the plausibility of our interpretation that the increases were due to the food assistance. And, third, sensitivity analyses on the main outcomes described in the statistical methods section, including the use of alternative matching variables and techniques, yielded similar results (not shown), supporting the internal validity of the study.

## Conclusions

We have demonstrated that food assistance programs can increase the physical component of HRQoL among ART naive PLHIV. Notably, in resource-poor settings such as Uganda, less than half of the individuals who qualify for ART under WHO guidelines receive it [68,70]. The findings are important because despite increased incorporation of food assistance components into HIV/AIDS programs, few studies have investigated the many potential benefits for PLHIV, including improvements in HRQoL, an alternative outcome and potential programmatic endpoint that is especially relevant given the chronic nature of HIV/AIDS.

The results should be particularly useful to programmers and policy makers because it was conducted within the routine program context of a large HIV/AIDS care and treatment provider and in coordination with one of the largest providers of food assistance in Africa. These features give the study a degree of external validity that would not have been possible had the evaluation substantially altered the provider’s standard operational design [71]. Efforts to address food insecurity within HIV programs should be expanded even further, and future research should examine whether in different contexts these types of programs can affect other psychosocial outcomes, including mental health components of HRQoL.

There are demonstrated links between food security and ART adherence and between ART treatment and HRQoL [25,33,67,72]. Moreover, improvements in HRQoL due to enhanced food security may themselves have feedback effects on self-care behavior, including ART adherence itself, further improving HRQoL [73]. Therefore, examining the effects of food assistance on HRQoL for PLHIV initiating or on ART would be another valuable extension of this study.

## Supporting Information

S1 Data Source data. (ZIP)

## Abbreviations

(ART): Antiretroviral therapy
(ATT): average treatment on the treated
(BMI): body-mass index
(g): grams
(HRQoL): Health-related quality of life
(HFIAS): Household Food Insecurity Access Scale
(MHS): mental health summary score
(MOS-HIV): Medical Outcomes Study HIV Health Survey
(PLHIV): People living with HIV/AIDS
(PHS): physical health summary score
(SD): standard deviation
(TASO): The AIDS Support Organization
(WFP): World Food Programme
(WHO): World Health Organization
